# High-throughput detection of clinically targetable alterations using next-generation sequencing

**DOI:** 10.18632/oncotarget.15875

**Published:** 2017-03-03

**Authors:** Julie A. Vendrell, David Grand, Isabelle Rouquette, Valérie Costes, Samira Icher, Janick Selves, Marion Larrieux, Aurore Barbe, Pierre Brousset, Jérôme Solassol

**Affiliations:** ^1^ CHU Montpellier, Arnaud de Villeneuve Hospital, Department of Pathology, Montpellier, France; ^2^ Department of Pathology, Institut Universitaire du Cancer Toulouse Oncopole, CHU de Toulouse, Toulouse, France; ^3^ IRCM, Institut de Recherche en Cancérologie de Montpellier, Montpellier, France; ^4^ INSERM, Montpellier, France; ^5^ Laboratoire d’excellence Labex TOUCAN, Toulouse, France; ^6^ Université de Montpellier, Montpellier, France

**Keywords:** NGS cancer panel, molecular diagnosis, targeted therapies, routine practice

## Abstract

Next-generation sequencing (NGS) has revolutionized the therapeutic care of patients by allowing high-throughput and parallel sequencing of large numbers of genes in a single run. However, most of available commercialized cancer panels target a large number of mutations that do not have direct therapeutic implications and that are not fully adapted to low quality formalin-fixed, paraffin-embedded (FFPE) samples. Here, we designed an amplicon-based NGS panel assay of 16 currently actionable genes according to the most recent recommendations of the French National Cancer Institute (NCI). We developed a panel of short amplicons (<150 bp) using dual-strand library preparation. The clinical validation of this panel was performed on well-characterized controls and 140 routine diagnostic samples, including highly degraded and cross-linked genomic DNA extracted from FFPE tumor samples. All mutations were detected with elevated inter-laboratory and inter-run reproducibility. Importantly, we could detect clinically actionable alterations in FFPE samples with variant allele frequencies as low as 1%. In addition, the overall molecular diagnosis rate was increased from 40.7% with conventional techniques to 59.2% with our NGS panel, including 41 novel actionable alterations normally not explored by conventional techniques. Taken together, we believe that this new actionable target panel represents a relevant, highly scalable and robust tool that is easy to implement and is fully adapted to daily clinical practice in hospital and academic laboratories.

## INTRODUCTION

The concept of personalized and targeted therapies has become a reality of practice for medical oncologists with the administration of specific molecules according to somatic genetic alterations [1, 2]. Comprehensive characterization of mutations in clinically actionable genes and key cancer pathways have become helpful in prognostic prediction and for guiding the selection of therapy, ultimately accelerating the development of personalized treatment [3].

Thus, European guidelines for non-small-cell lung cancer (NSCLC) management encourage a wide coverage of broader molecular profiling including conventional mutations such as exons 18-21 *EGFR* as well as rarer driver events for which specific drugs may already be available or accessible through clinical trials [4]. Until recently, indications for standard-of-care molecular testing in colorectal carcinomas (CRC) included testing for exon 2 *KRAS* mutational status as a predictor of response to cetuximab (Erbitux; Merck KGaA) and to panitumumab (Vectibix; Amgen Inc.) [5]. Now, guidelines recommend that exon 3 and exon 4 *KRAS* and *NRAS* mutation status should also be determined [6]. This change illustrates that the number (or the extent) of biomarkers that will need to be assessed in clinical daily practice in molecular pathology is rapidly increasing. This increase requires the implementation of methods probing the detection of multiple genes and mutations, including rarely encountered or unexpected genetic variations. Moreover, this increase in the number of genes to be tested is associated with a decrease in the number of analyzable samples and a need to optimize technical procedures. Finally, as the number of clinically relevant genetic variants has increased, routine laboratory tests have evolved, moving from single mutations to multiplex hotspot evaluations in multiple cancer genes.

Next-generation sequencing (NGS) has transformed the cancer genomics landscape by enabling comprehensive cancer genome characterization of unprecedented scope [7, 8]. NGS allows for the simultaneous, massive parallel detection of hundreds to thousands of recurrent somatic mutations and high-throughput sample processing. Several NGS approaches have been successfully clinically developed in oncology, such as amplicon–based panels, capture–based panels or whole-genome sequencing [9]. PCR-based technology kits are commercially available [10, 11]. However, even if the use of these kits is possible in clinical laboratories, they are not systematically adapted for the molecular diagnosis of solid tumors in the clinical setting. Indeed, the size of the amplicons are generally too long (~180 bp) for amplification of some highly degraded and cross-linked genomic DNA extracted from formalin-fixed, paraffin-embedded (FFPE) tumor samples, reducing the chance for these patients to benefit from the contribution of NGS in diagnosis. Moreover, most of the available panels are not specifically dedicated to current actionable genomic mutations.

In 2006, the French National Cancer Institute (NCI) funded a nationwide program for the systematic routine analysis of genomic mutations. This program is based on the establishment of a network of 28 certified molecular genetic platforms [12]. Located in public hospitals, each platform is expected to offer free molecular testing to both private and public centers of a specific geographic area for the local population [13]. The main goals of this organization are to assure the access of all patients to available targeted therapies and drug innovations and to promulgate guidelines for clinical laboratory analyses [14]. Additionally, the French NCI is responsible through recommendations provided by an external panel of experts for the implementation of new biomarkers. Thus, recently, a limited checklist covering selected biomarkers from 16 cancer-related genes with relevance across a broad scope of solid tumors have been provided, to identify: (i) alterations related to approved drugs that directly or indirectly target genes; (ii) alterations that predict resistance to existing treatments; and (iii) alterations for which a molecule in phase II-III clinical trials is available (www.e-cancer.fr).

In the present study, we assess reliability and accuracy of a Diagnostic Solid Tumor Panel (DSTP), a new 48-target amplicon-based NGS assay specifically dedicated to the detection of clinically actionable genetic alterations that are critical to cancer care. We identified and validated molecular alterations in a broad array of clinical samples in two independent molecular platforms and demonstrated that DSTP is a scalable multiplexing solution compatible with routine turnaround time that has been adapted to highly degraded samples with a simple amplicon-based procedure.

## RESULTS

### DSTP development and workflow description

**Figure 1:**
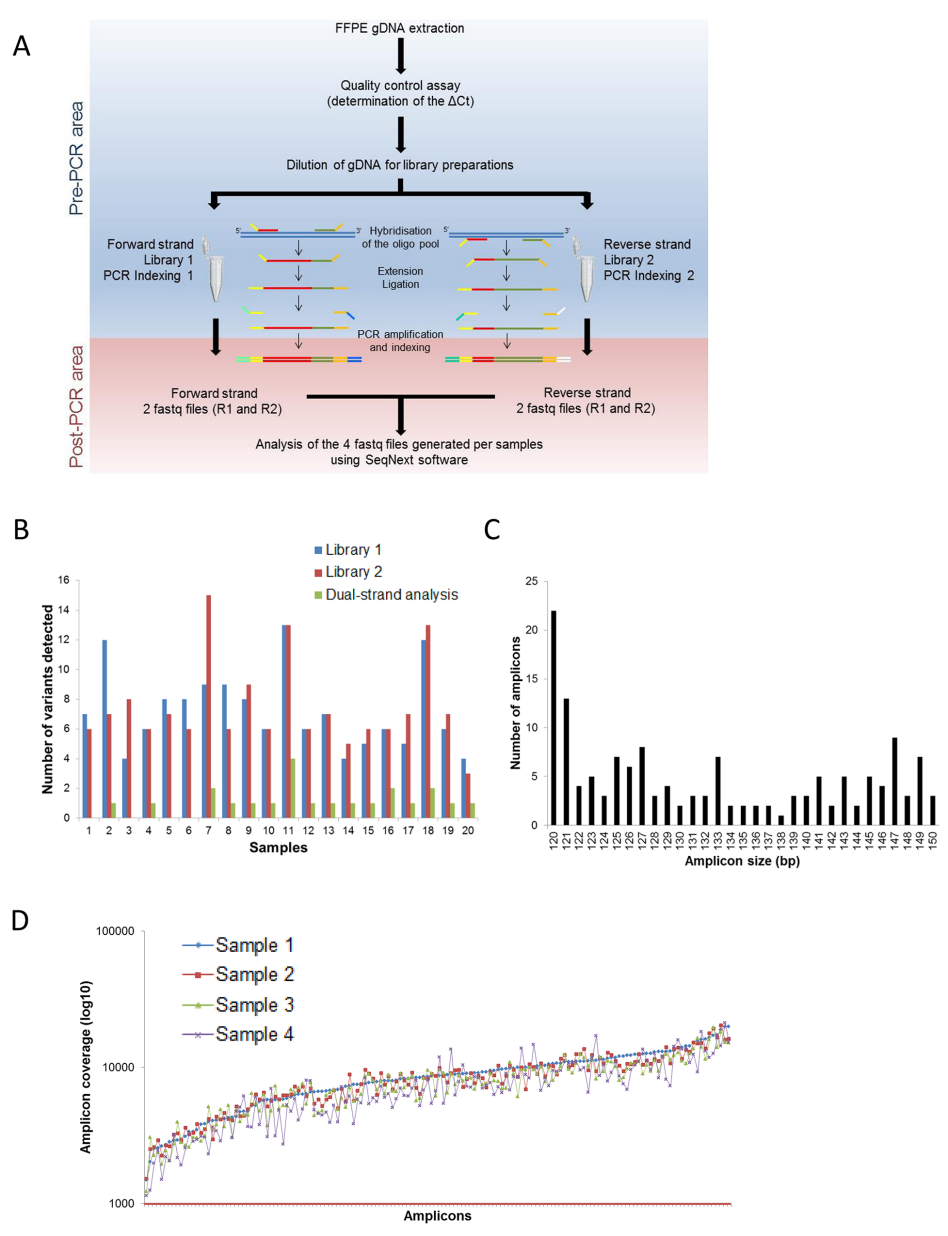
Process workflow and specifications of the DSTP **A**. Description of the DSTP library preparation workflow. **B**. Interest of the dual-strand library preparation to detect mutations. Number of mutations detected using the information from Library 1 only (blue histogram), Library 2 only (red histogram) or from both libraries (green histogram). **C**. Amplicon size distribution for the 150 amplicons of the panel. **D**. Coverage distribution plot of 4 representative samples sequenced in a run with 32 samples pooled on a v2 standard flow cell. The Red line represents the targeted depth (1000×).

To minimize fixation DNA damage that can potentially misclassify modified bases and generate artifacts, we used a dual-strand library preparation approach that generates specific libraries for each of the two strands of DNA (Figure 1A). This workflow resulted in two independent libraries per sample that were analyzed independently. The information obtained was bioinformatically combined to distinguish true variants from artifacts by reporting only those that are commonly detected on both strands of DNA (Figure 1B).

To circumvent the problem of analyzing highly degraded FFPE samples, we developed a panel with an amplicon median size of 130 bp (minimum: 120 bp; maximum: 150 bp; Figure 1C). This panel specifically targets 48 specified exons of 16 genes (Table 1) covered by 150 amplicons. Oligos were carefully designed to hybridize in regions with <1% SNP described in different genomic databases, to have a low % GC and a median size of 27 bases (min: 22; max: 30). After trimming primers, the minimal padding into the intronic region flanking the exons was -7 and +6, allowing for the detection of mutations in splicing sites. The minimum overlap between different amplicons was 1 base. The sequenced size of the panel was approximately 20 Kb per libraries corresponding to 40 Kb per samples, enabling the analysis of a larger number of samples per run using a v2 MiSeq Sequencing Chemistry flow cell with a read depth per amplicon superior to 1000x analysis for a run analysis of 32 samples (Figure 1D).

**Table 1:** The French National Cancer Institute recommendations for the detection of mutations in solid tumor samples

Gene	Transcript ofreference	Exons (hotspots)	Associated molecules	Accessibility totherapy
*AKT1*	NM_001014431.1	3	AKT inhibitors	Clinical trials
*ALK*	NM_004304.1	23, 24, 25	crizotinib, ALK inhibitors	AcSé program, Clinical trials
*BRAF*	NM_004333.4	11, 15	vemurafenib, dabrafenib	EMA approval
*EGFR*	NM_005228.3	18, 19, 20, 21	anti-EGFR	EMA approval
*ERBB2 (HER2)*	NM_004448.2	20	trastuzumab, neratinib	Clinical trials
*ERBB4*	NM_005235.2	10, 12 (E452K and R393W)	Afatinib	Clinical trials
*FGFR2*	NM_000141.4	7, 12, 14 (S252, N549, K659)	FGFR inhibitors	Clinical trials
*FGFR3*	NM_000142.4	7, 9, 14 (R248 to S249 and G370 to Y373)	FGFR inhibitors	Clinical trials
*HRAS*	NM_005343.2	2, 3, 4	MEK inhibitors	Clinical trials
*KIT*	NM_000222.2	8, 9, 11, 13, 17, 18	imatinib	EMA approval
*KRAS*	NM_033360.2	2, 3, 4	panitumumab, cetuximab	EMA approval
*MAP2K1 (MEK1)*	NM_002755.3	2	MEK inhibitors	Clinical trials
*MET*	NM_001127500.1	2, 14, 15, 16, 17, 18, 19, 20	crizotinib	AcSé program
*NRAS*	NM_002524.3	2, 3, 4	panitumumab, MEK inhibitors, BRAF inhibitors	EMA approval, Clinical trials
*PDGFRA*	NM_006206.4	12, 14, 18	imatinib	EMA approval
*PIK3CA*	NM_006218.2	10, 21	PI3K inhibitors	Clinical trials

EMA, European Medicines Agency

### Determination of the DSTP sensitivity and reproducibility

**Figure 2:**
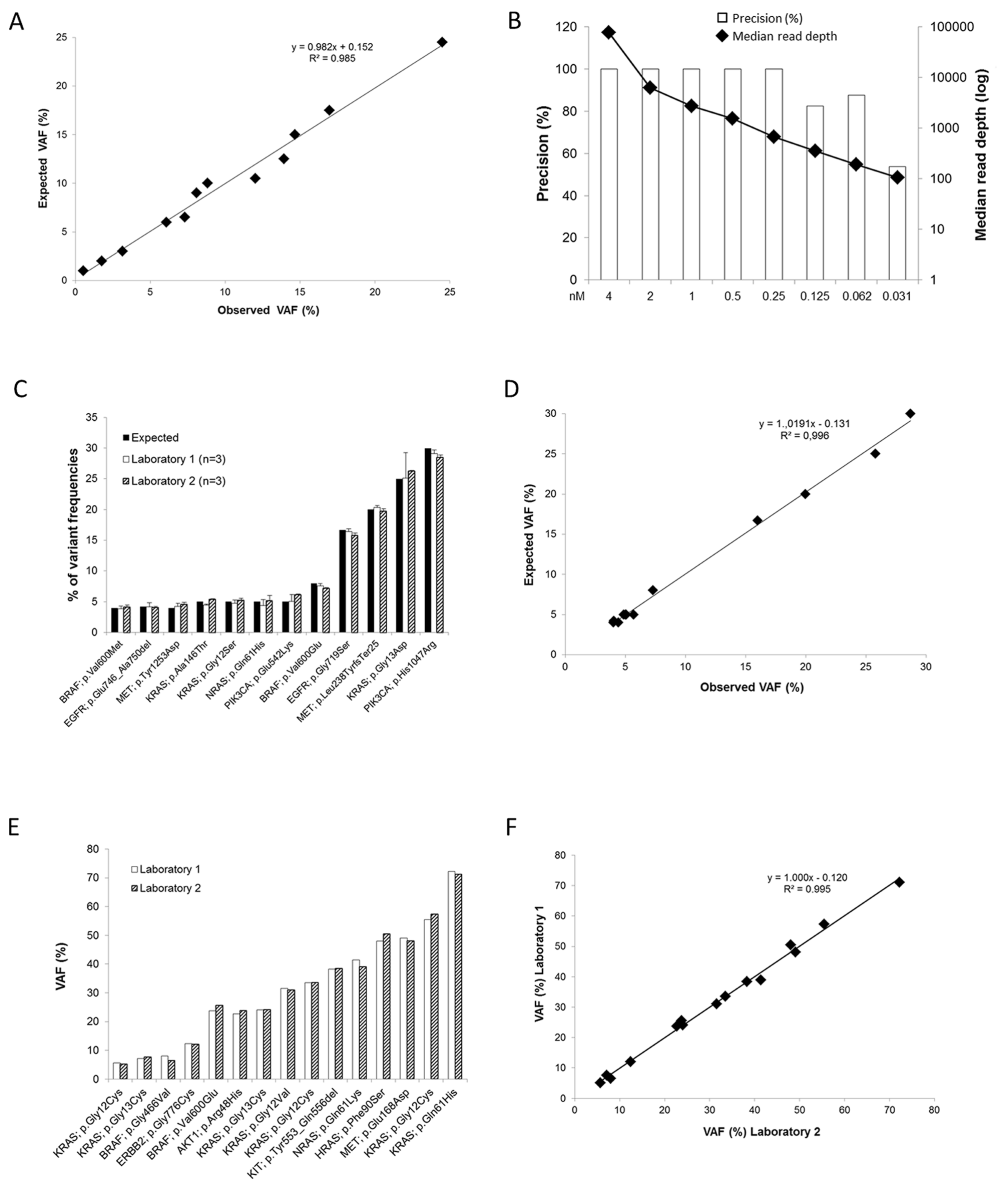
Sensitivity and reproducibility of the DSTP **A**. Correlation between the expected and the observed VAF (%) for the FFPE Quantitative Multiplex control sample. Observed values are means of 3 independent experiments. **B**. Representation of the precision according to the read depth using serial dilutions of the FFPE Quantitative Multiplex control sample. **C**. For the Tru-Q NGS DNA 3 control sample, concordance between the percentages of VAF measured by droplet PCR (black bars) and by NGS using the DSTP in two independent laboratories (white and dashed bars). The results from each laboratory are expressed as the mean ± standard deviation (SD) of three independent experiments. **D**. Correlation between the expected and the observed VAF (%) for the Tru-Q NGS DNA 3 control sample. Observed values represent means of 6 independent experiments performed in the two laboratories. **E**. Using clinical samples extracted from FFPE, inter-laboratory reproducibility of 15 mutations present in the samples. **F**. Correlation between the VAF (%) reported by the two laboratories for the clinical samples.

To test the sensitivity of our DSTP, we sequenced a quantitative multiplex reference standard control sample extracted from an FFPE block. This control sample included a range of engineered and endogenous gene variants present at precise allelic frequencies and fully characterized by droplet digital PCR (Supplementary Table 1). It has been selected to cover a broad range of mutations targeted by the panel (point mutations, deletion) with predicted frequencies as low as 1%. Interestingly, all the expected mutations were detected, even very low variant frequency *EGFR* mutations (T790M 1%, ΔE746-A750 2%, L858R 3%) (Figure 2A and Supplementary Table 1). The inter-run reproducibility was also tested using this sample and an excellent and significant correlation between expected and observed variant frequencies was observed (R^2^ = 0.985; r = 0.993; Pearson correlation test; *P* < 10^-4^; Figure 2A). Finally, to determine the precision of our DSTP according to the read depth, different libraries were prepared and normalized to concentration ranging from 4 nM to 0.03 nM. After sequencing, all the mutations present in the control sample could be detected even for libraries highly diluted; however the number of false positive increased with the dilution (Figure 2B and Supplementary Table 2). Altogether, these results allowed us to define a median read depth cutoff of 700x demonstrating a complete concordance with the expected results.

To further assess the inter-run and inter-laboratory reproducibility, a quantitative multiplex DNA reference sample (Tru-Q NGS DNA 3) with predicted frequencies as low as 4% was sequenced in each run performed by two independent laboratories: the University Hospital of Montpellier - Montpellier Cancer Institute (laboratory 1) and the University Hospital of Toulouse (laboratory 2). As expected, all mutations were detected in each experiment (Figure 2C and Table 2), including mutations with low variant allele frequency - VAF (from 4% to 5%, n = 7). Moreover, an excellent and significant correlation (R^2^ = 0.996; r = 0.996; Pearson correlation test; *P* < 10^-4^; Figure 2D) was observed between the expected and the observed VAF (mean of 6 independent experiments). Because this quantitative multiplex DNA reference sample corresponds to a commercial non-degraded genomic DNA, we further explored the inter-laboratory reproducibility with clinical samples extracted from FFPE tissues. For this purpose, 20 FFPE tissue samples were blindly analyzed by the two laboratories. Among these samples, 7 were reported non-mutated by the two laboratories, validating the results obtained by Sanger. Regarding the 14 remaining samples, 15 mutations previously detected by routine techniques were also identified using DSTP. Interestingly, the VAF reported by the two laboratories were highly similar and significantly correlated (R^2^ = 0.995; r = 0.998; Pearson correlation test; *P* < 10^-4^; Figures 2E-2F).

**Table 2:** Study of the inter-run and inter-laboratory reproducibility using the Tru-Q NGS DNA3 control sample

Gene	Nucleotide change	Amino Acid change	VAF (Laboratory 1)			VAF (Laboratory 2)			Mean inter-laboratories			ExpectedVAF (%)^b^
			Mean^a^	SD	CV(%)	Mean^a^	SD	CV(%)	Mean^a^	SD	CV(%)	
*BRAF*	c.1798G>A	p.Val600Met	3.88	0.45	11.55	4.10	0.37	9.09	4.01	0.37	9.16	4.0
*EGFR*	c.2235_2249delGGA ATTAAGAGAAGC	p.Glu746_Ala750del	4.13	0.70	16.98	4.01	0.23	5.69	4.06	0.39	9.64	4.2
*MET*	c.3757T>G	p.Tyr1253Asp	4.25	0.48	11.30	4.56	0.33	7.28	4.44	0.38	8.45	4.0
*KRAS*	c.436G>A	p.Ala146Thr	4.43	0.17	3.89	5.41	0.10	1.78	5.02	0.55	10.86	5.0
*KRAS*	c.34G>A	p.Gly12Ser	4.78	0.48	9.97	5.26	0.34	6.44	5.07	0.43	8.46	5.0
*NRAS*	c.183A>T	p.Gln61His	4.42	0.92	20.84	5.17	0.85	16.47	4.87	0.86	17.70	5.0
*PIK3CA*	c.1624G>A	p.Glu542Lys	5.07	1.09	21.50	6.13	0.13	2.12	5.71	0.80	14.05	5.0
*BRAF*	c.1799T>A	p.Val600Glu	7.57	0.39	5.17	7.13	0.17	2.42	7.30	0.33	4.56	8.0
*EGFR*	c.2155G>A	p.Gly719Ser	16.40	0.44	2.70	15.75	0.42	2.68	16.01	0.51	3.22	16.7
*MET*	c.710delT	p.Leu238TyrfsTer25	20.33	0.29	1.41	19.74	0.39	1.98	19.97	0.45	2.26	20.0
*KRAS*	c.38G>A	p.Gly13Asp	25.13	4.14	16.49	26.24	0.12	0.46	25.80	2.16	8.38	25.0
*PIK3CA*	c.3140A>G	p.His1047Arg	29.09	0.63	2.17	28.45	0.41	1.43	28.71	0.55	1.93	30.0

^a^ Mean of three independent experiments

^b^ measured by ddPCR

SD, Standard deviation; CV, Coefficient of variation; VAF, Variance allele frequencies; ddPCR, droplet digital PCR

### DSTP validation in a clinical molecular diagnosis cohort

**Figure 3:**
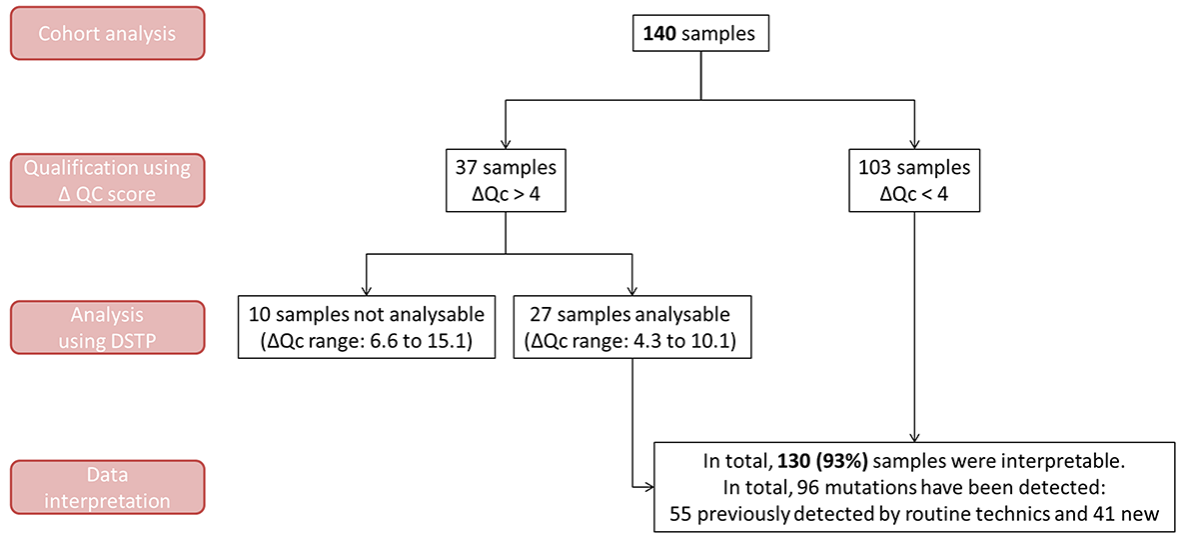
Number of analyzable and interpretable samples depending on the ΔQc scoring A ΔQc score was calculated for each sample before DSTP processing. Sample analyses and interpretations are presented according to their ΔQc score (ΔQc > or < 4). ΔQc < 4 are considered as not suitable for analysis using commercialized NGS panels.

Next, 140 samples from three different tumor locations (lung, colorectal, and skin; Table 3) that were previously analyzed by conventional methods for the research of actionable mutations were randomly selected, distributed to each laboratory and blindly analyzed by NGS using the DSTP. Before library preparation, a qPCR-based assay was performed to qualify the gDNA extracted from the archival FFPE tissues (determination of a ΔQc score). Usually, samples that exhibit a ΔQc > 4 are not suitable for NGS analysis. Because our panel was specifically designed to allow for the analysis of poor quality gDNA, all the samples present in our cohort have been sequenced, even those with a ΔQc > 4 that are usually not interpretable using NGS procedures (n = 38; 27%; Figure 3). Among them, 27 samples (71%) with a ΔQc range of 4.3 to 10.1 were successfully sequenced and only 10 samples (29%) (ΔQc range: 6.6 to 15.1) were considered to be not interpretable with our DSTP. Among the 130 analyzable samples, a total of 96 non-synonymous somatic mutations were detected by DSTP, 87 with single nucleotide variants (SNV), 4 multi nucleotide variants (MNV), and 5 with insertion/deletions (indels) (Figure 3 and Supplementary Table 3). All alterations previously reported with our routine methods were detected with the DSTP analysis, including the 27 samples with a ΔQc range of 4.3 to 10.1 (Figure 3, and Supplementary Table 3).

**Table 3:** Patient and specimen characteristics

Characteristics	Lung cancer samples(n = 77)		CRC samples (n = 41)GIST samples (n = 6)		Melanoma samples(n = 16)	
	n	%	n	%	n	%
**Sex**						
Male	42	54.5	18	38.3	11	68.8
Female	35	45.5	29	61.7	5	31.3
**Type of specimen**						
Biopsy	43	55.8	24	51.1	9	56.3
Surgical specimen	32	41.6	22	46.8	7	43.8
Unknown	2	2.6	1	2.1	0	0.0
**Tumor sites**						
Primary tumor	51	66.2	38	80.9	9	56.3
Metastases	22	28.6	6	12.8	7	43.8
Peritoneal carcinomatosis	1	1.3	3	6.4		
Unknown	3	3.9				
**Tissue preparation**						
Frozen	17	22.1	5	10.6	2	12.5
Fixed	62	80.5	42	89.4	14	87.5
Formalin	38	49.4	33	70.2	2	12.5
AFA fixative	4	5.2	3	6.4	12	75.0
RCL2^®^	1	1.3	6	12.8	0	0.0
Unkown	2	2.6	0	0.0	0	0.0
**Tumor cell content**						
*<50%*	9	11.7	10	21.3	3	18.8
≥50%	46	59.7	34	72.3	13	81.3
Unkown	22	28.6	3	6.4	0	0.0
**NGS process**						
Analysable	68	88.3	46	97.9	16	20.8
Not analysable	9	11.7	1	2.1	0	0.0

AFA, alcohol, formalin, and acetic acid

We then assessed the performance of DSTP. A total of 53,534 nucleotides were commonly interrogated by both NGS and conventional technical approaches; 135 were variants and 53,399 were wild (Table 4). DSTP exhibited a sensitivity of 100% (95% Confidence Interval 97.2% - 100%), a specificity of 100% (95% Confidence Interval 99.99% - 100%), and an accuracy of 100% (95% Confidence Interval 99.99% - 100%), underlying the excellent concordance between both approaches (Table 4). Finally, we compared the results obtained by our panel to those acquired with a commercial NGS panel. For this purpose, the quantitative multiplex DNA reference sample and 5 FFPE samples were sequenced with the commercial TruSight Tumor library preparation kit, which also uses a double-stranded library preparation approach. An excellent and significant concordance could be observed between both panels in the % of VAF measured for the control and the clinical samples (R^2^ = 0.995, r = 0.997; Pearson correlation test; *P* < 10^-4^) (Supplementary Table 4 and 5).

**Table 4:** Method correlations between the DSTP and routine techniques

	DSTP
**Number of bases sequenced^a^**	53534
True Positive (TP)	135
True Negative (TN)	53399
False Positive (FP)	0
False Negative (FN)	0
**Performance**	
Sensitivity	100%
Specificity	100%
Accuracy	100%
Precision	100%

^a^ Number of bases interrogate by both DSTP and routine techniques

### DSTP improved the mutation detection rate in clinical samples

**Figure 4:**
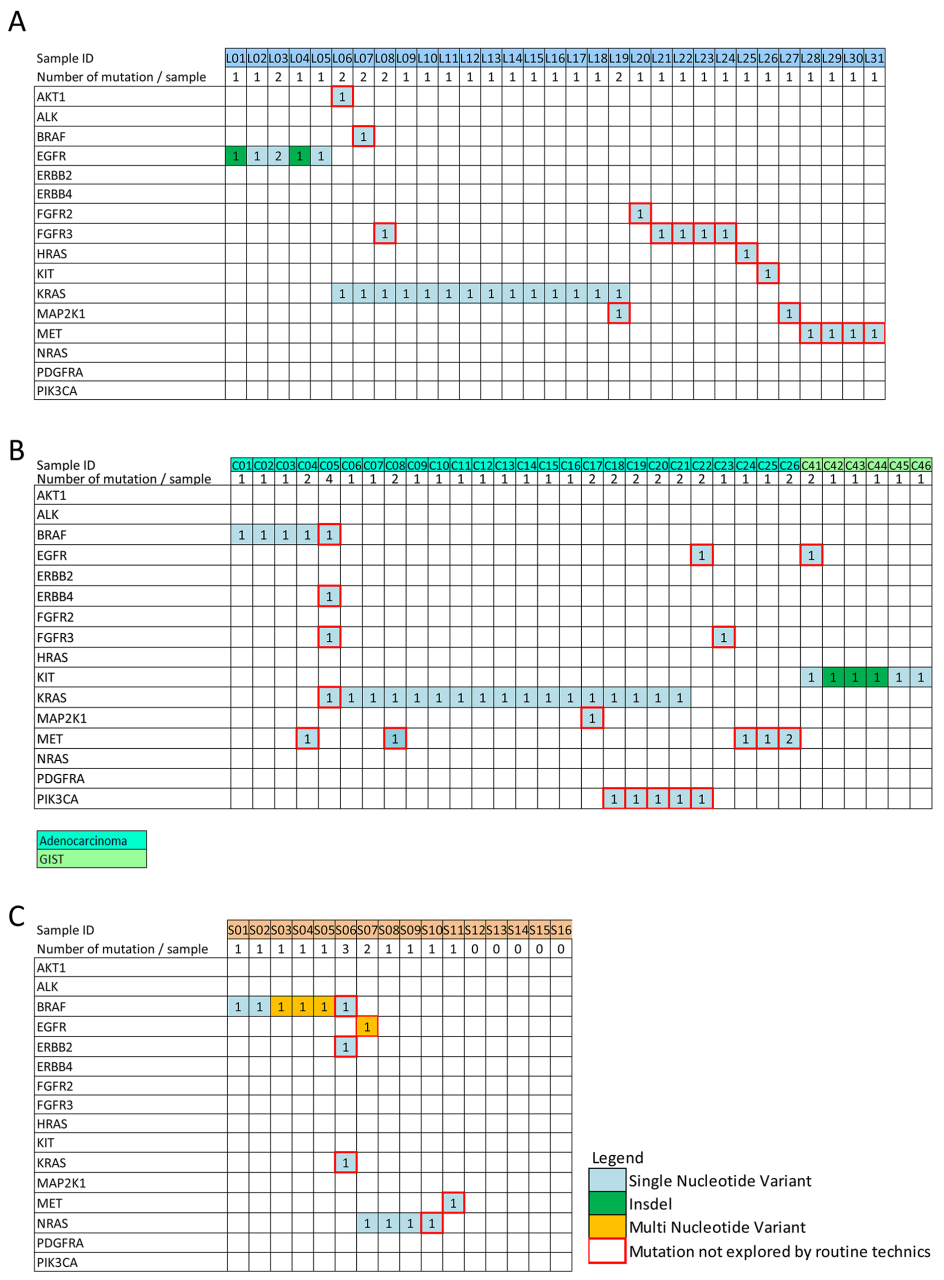
Somatic mutations detected among the 130 tumor samples analyzable with the DSTP Data are shown for **A**. lung cancer, **B**. colon cancer and **C**. skin cancer. DSTP detected 95 non-synonymous somatic mutation variants in the 130 analyzable clinical samples, and confirmed the 55 alterations previously detected by routine techniques. Only samples for which at least one mutation has been detected are illustrated. Numbers in the square represent the number of mutations detected for the corresponding gene. The Red squares represent mutations detected using DSTP that were not targeted by routine techniques.

**Figure 5:**
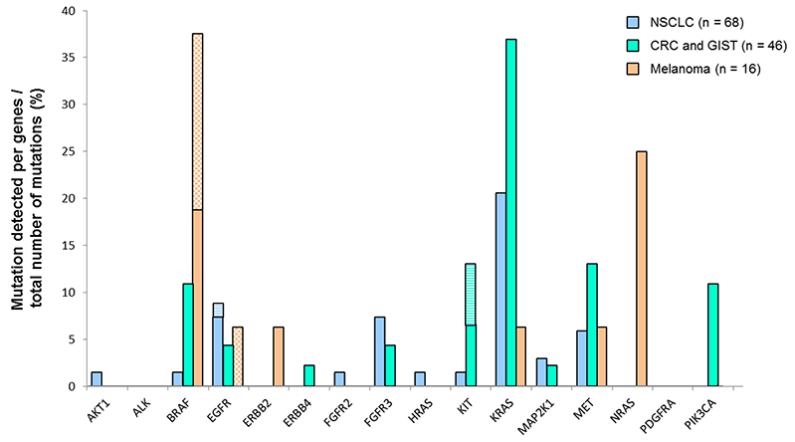
Gene mutations detected according to the tumor localization The results are expressed as percentage of mutations detected by gene out of the global number of detected mutations per the different cancer types. Plane histograms, SNV mutations; checked histograms, MVN mutations; striped histograms, indels.

When considering the samples that are routinely encountered, the use of DSTP allowed the detection of 41 mutations that were not normally recognized by conventional techniques. Notably, 21 samples with no alterations detected by HRM and/or Sanger were determined to harbor one or more actionable mutations by DSTP (Table 5 and Supplementary Table 3). Moreover, additional mutations could also be detected for 13 samples that already presented one mutation detected by routine analysis (Figure 4). Briefly, among newly detected mutations in lung adenocarcinoma, *AKT, BRAF, FGFR2, HRAS*, and *KIT* mutations were detected in one sample (1.3%), *MAP2K* in two samples (2.6%), *MET* in 4 samples (5.9%), and *FGFR3* in 5 samples (7.4%) (Figure 5 and Supplementary Table 3). In CRC, an uncommon *BRAF* (c.1742A>T, p.Asn581Ile) mutation and a *KRAS* (c.57G>T; p.Leu19Phe) mutation were found in one sample (2.4%). *EGFR, ERBB4*, and *MAP2K1* mutations were found in one sample (2.4%), an *FGFR3* mutation in 2 samples (4.9%), and a *PIK3CA* or *MET* mutation in 5 samples (12.2%) (Figure 5 and Supplementary Table 3). In GIST, only one sample (16.6%) exhibited a new non-studied *EGFR* mutation (c.2230A>G; p.Ile744Val). Thus, in melanoma, one sample (6.2%) had an uncommon *BRAF* (c.1400C>T; p.Ser467Leu) mutation, one (6.2%) had an *NRAS* (c.385C>T; p.Gln129*) mutation, and rare *EGFR, ERBB2, KRAS*, and *MET* mutations were also exhibited in one sample (6.2%). Finally, using DSTP, we were able to simultaneously detect multiple relevant somatic loci for 16 specimens (12.3%) (Figure 4). The corresponding allelic frequencies are given in Supplementary Table 2. Of note, all these mutations were further validated by newly specifically designed HRM, Sanger or pyrosequencing (data not shown).

**Table 5:** Improvement of the mutational rate detection using DSTP

	DSTP	Routine techniques
Global number of mutations detected	96	55
Patient diagnosis		
Mutated	74	53
WT	56	77

Therefore, if we take into consideration all the mutations detected in these samples, the overall molecular diagnosis rate was 40.7% using conventional techniques and 59.2% using our NGS panel, demonstrating the need to increase the accessibility of an actionable NGS panel in routine clinical practice for solid tumor sample management.

## DISCUSSION

Given the increasingly critical role of molecular investigations in the management of cancer patients, there is an immediate need for robust, high-quality diagnostic tests. NGS technologies have revolutionized genomic medicine and the therapeutic care of patients by allowing simultaneous, high-throughput and parallel sequencing of a large number of genes without the need to perform several sequential tests [9]. Several studies have demonstrated the clinical benefit of using NGS in hospital laboratories. Indeed, the NGS approach is sensitive and cost-effective with a short turnaround time, and it uses a comparatively small quantity of DNA versus conventional PCR methods such as Sanger sequencing that remain the gold standard [15, 16]. However, the complexities of this method and the high volume of data generated by each run have clearly slowed the spread of its availability in molecular pathology laboratories [17, 18]. Therefore, molecular academic diagnostic laboratories need to enhance their molecular methods through robust and optimized solutions that can be set up in a routine, daily activity and readily comply with clinical performance, staff expertise, quality and guideline standards.

Targeted amplicon-based library preparation methods combined with parallel sequencing offered a relevant solution [19]. Torrent AmpliSeq Colon & Lung Cancer Panel (Life Technologies) and TruSight Tumor Panel (Illumina) are commercial panels that target actionable cancer-related genes (11 and 26, respectively). These panels have been clinically validated and offered as a clinical test by several institutions [10, 20–22]. However, Torrent AmpliSeq Colon & Lung Cancer is restricted to colon and lung tumors only and is, therefore, not available for alterations associated with other types of cancers (e.g., c*KIT* for melanoma and GIST or *PDGFRA* for GIST are not covered). Moreover, this assay is also not adapted to umbrella trials [23], where patients with a type of morphologically defined cancer are assigned to a treatment arm on the basis of the genetic mutations detected in their tumor. Several genes such as *STK11*, *FOX22*, *PTEN*,*CDH1* or *TP53*, which are included in both of these commercial panels are not useful because they do not have any clinical applications to date, thereby reducing the number of patients who can be analyzed in one flow cell. In addition, larger commercially available panels have been validated on FFPE tumor samples [24–27]. Finally, Hovelson *et* *al*. recently developed a large DNA- and RNA-based panel that allows the detection of point mutations, indels, gene fusions and copy number variations in a single experiment [24].

In this study, we detailed the clinical validation of a new targeted amplicon-based NGS assay for the detection of clinically relevant somatic variants in routine FFPE tumor samples, according to the last French NCI recommendations. We sequenced specific exons of 16 cancer-related genes, including the most clinically relevant genes such as *KRAS, NRAS, EGFR, BRAF*, c*KIT*, as well as other targetable genes that are known to be somatically altered in solid cancers based on recent scientific and clinical literature. A total of 140 FFPE samples covering NSCLC, CRC, GIST and melanoma specimens were assessed. When using an FFPE control sample harboring well-characterized mutations, DSTP allows the reproducible detection of single base mutations present at 1% and deletions at 2%. However, each laboratory should determine their own VAF detection cut-off through the realization of a series of independent assays. Moreover, DSTP results demonstrated an excellent overall performance. Indeed, DSTP detected all expected SNVs, MNVs, indels, and wild-type loci highlighted by orthogonal methods (HRM/Sanger sequencing/pyrosequencing/ddPCR), indicating 100% concordance for known variants. In regard to these data, using a VAF cut-off of 5% with a median coverage of 500-fold may allow unambiguous detection of mutations. Finally, we observed a high genotyping success rate of 92.9%, with a 73.0% rescue of successfully analyzed samples by this approach compared to the TruSight Tumor kit. A recent study [21] supported this finding, as they reported that only 47% and 0% of samples with a ΔQc > 4 and a ΔQc > 6, respectively, could be correctly sequenced using the TruSight Tumor kit, whereas with DSTP, 73% of samples with a ΔQc value > 4.0 and 44% of those with ΔQc value > 6.0 were correctly sequenced. This can be attributed to our short amplicon design. With a maximum amplicon size of 150 bp, DSTP is, to our knowledge, the shortest designed panel to date, maximizing its clinical usefulness in routine practice. Of note, melanin contamination in highly pigmented melanoma samples has been shown to potentially inhibit PCR amplification [28]. Using our technical procedure, all melanoma samples, including highly contaminated samples were successfully analyzed (data not shown). Moreover, our workflow procedure also minimized the cross contamination risk between samples because no amplification is processed prior to indexing.

As a demonstration of the clinical utility of DSTP in a routine setting, in addition to conventional *EGFR*,*KRAS*, and *BRAF* alterations, we identified a significant number of additional mutations with potential clinical impact for 34 patients. Indeed, preclinical and clinical data support the argument that patients harboring these mutations could potentially benefit from specific therapeutic trials, such as BOLERO-1 and BOLERO-3 trials in breast cancer patients with *PIK3CA* mutations [29]. In addition, DSTP could detect mutations at very low allelic frequencies (<10%). This high sensitivity is especially important for *EGFR* T790M or *EGFR* C797S mutations because quantitative assessment by targeted NGS could enable early prediction of acquired resistance to first, second or third generation of EGFR tyrosine kinase inhibitors [30]. The most practical clinical benefit of DSTP testing was the ability to assess multiple relevant somatic loci simultaneously. Recently, increased synergistic activities of drug combinations have been observed. An association between MEK and Aurora-A kinase inhibitors induced a higher antitumor response in CRC harboring concomitant *KRAS* and *PIK3CA* mutations [31]. Clinical trials of dual targeted inhibition of MEK and the PIK3CA/AKT/mTOR pathway in patients with concomitant mutations of genes belonging to these pathways are also underway (for review, [32]). In our study, 15 samples harbored double mutations, 9 of them with concomitant mutations in genes from the PI3K/AKT/mTOR and RAS/RAF/MEK pathways, suggesting that the corresponding patients may be included in clinical trials and benefit from dual targeted therapy. Moreover, 2 specimens presented 3 and 4 concomitant mutations. Although the significance of these concomitant mutations remains unknown, the prognostic significance of these simultaneous mutations as well as its impact in terms of progression-free or overall survival should be better studied in larger cohorts in the future [33]. Finally, the most important clinical impact of using a multigene cancer panel is the ability to assess multiple relevant somatic loci simultaneously, helping in the identification of a subset of patients who would most benefit from alternative single or dual pathway inhibition therapy [34]. NSCLC patients harboring rearrangements within the *ALK*, *ROS1* and *RET* gene can be targeted in lung adenocarcinoma using specific therapies such as Crizotinib. Although our panel was not designed to detect such alterations, specific novel technologies with the ability to simultaneously detect *ALK*,*ROS1* and *RET* fusions in a single assay show promise for use in clinic.

Formaldehyde fixation processes have been largely found to induce strand breakage and reversible or irreversible chemical modifications, such as cytosine deamination and cyclic base derivatives that impair PCR amplification [35]. Moreover, PCR enrichment steps also represent a possible source of base-composition bias in library preparation, adding an extra level of artifacts and technology bias, and thus challenging bioinformatics analysis for clinical applications of low frequency variant calling [36, 37]. Therefore, NGS sequencing using gDNA extracted from FFPE samples is highly challenging because DNA is degraded and limited amounts of sample are usually available. To circumvent these problems, we have developed a dual-strand library preparation methodology based on the bidirectional sequencing of the positive and negative DNA strands in two independent library preparations per sample. Notably, this approach markedly reduces the possibility of both false-negative and false-positive results. Indeed, we observed a concordance of 100% between DSTP and routine approaches with no false positive mutations observed in our series.

Taken together, our results demonstrate the high sensitivity and specificity of the approach described herein, as all the mutations harbored by the samples studied have been detected with important inter-laboratory and inter-run reproducibility. Moreover, it is important to note that detection of clinically actionable alterations in FFPE samples with VAF as low as 1% could be detected with high confidence. Finally, DSTP represents a highly scalable approach, commercialized by Illumina, that can be easily implemented into clinical daily practice and future French NCI precision medicine trials, demonstrating that DSTP represents a new relevant clinical tool for a higher level of therapeutic care for patients.

## MATERIALS AND METHODS

### Tumor samples and DNA extraction

This study was performed with approval from the Institutional Review Board and in concordance with regulatory guidelines regarding clinical assay validation. FFPE tissues from lung cancer, colon cancer and melanoma that have been submitted to the University Hospital of Toulouse or Montpellier (France) for mutation analysis performed by high resolution melting (HRM) analysis and Sanger sequencing were included in this study (samples from 2012 to 2015). Table 1 lists the characteristics of the patients and the corresponding specimens enrolled in the study. Before extraction, all lesions were excised and submitted for pathological examination using standard techniques. The percentage of tumor cells in the series ranged from 10-100%. Tissue punches using a 1 mm needle or 10-μm thick section were performed from tumor paraffin blocks. DNA extraction was performed using the QIAamp DNA FFPE Tissue kit (Qiagen) or the Maxwell^®^ 16 FFPE Plus LEV DNA Purification Kit (Promega) according to the manufacturers’ recommendations. Some fresh frozen tumor samples were also included (n = 24) and extracted using the QIAamp DNA Mini kit (Qiagen). Extracted DNA was quantified using a NanoDrop spectrophotometer (Thermo Scientific, Wilmington, USA) or the Qubit dsDNA broad range assay kit in combination with a Qubit Fluorometer (Thermo Scientific). A qPCR-based assay was also performed to qualify the extracted genomic DNA (gDNA) samples.

### Custom amplicon panel design

Specific exons of 16 clinically relevant cancer genes were selected for targeted sequencing (Table 2). Amplicon design was performed by the Illumina Concierge team according to the following recommendations: amplicon size should not exceed 150 bp, 100% on-target sequence coverage (Table 2), as well as a minimum of 5 bp exon padding. Following all these constraints, the theoretical on-target panel size is 8.87 Kb.

### Library preparation, sequencing and bioinformatics analyses

Experiments were performed in two independent clinical laboratories: the University Hospital of Toulouse and the University Hospital of Montpellier (France). The gDNA samples extracted from FFPE samples were previously qualified by comparing the amplification efficiency for the FFPE samples to a standard DNA, according to the manufacturer's recommendations. A ΔQc was then calculated and used to determine the fold dilution required for library preparation (see Supplementary Materials and Methods for further details). For each sample, two libraries were generated using two independent oligo pools containing upstream and downstream oligos specific to the forward and reverse strand of the targeted regions of interest (Figure 1A). Library preparation consisted of the hybridization of the oligo pool, a purification step to remove unbound oligos, and an extension-ligation step at 37°C for 45 minutes, resulting in the formation of products containing the targeted regions of interest flanked by sequences required for the amplification. These products were then amplified using primers that add index sequences for sample multiplexing (i5 and i7) and common adapters required for cluster generation (P5 and P7) (Figure 1A). PCR products were purified using AMPure XP beads (Beckman Coulter, Brea, CA), quantified, normalized to 4 nM to ensure equal library representation in the pooled sample and sequenced on an MiSeq instrument (Illumina) (Figure 1A). After pair-end sequencing (2×100 cycles), the four FastQ files generated per sample by the MiSeq Reporter software (Illumina) were analyzed using SeqNext software (JSI Medical Systems, Ettenheim, Germany). Filters used for the variant calling are detailed in Supplementary Materials and Methods. Non-template control, ACD1 (control sample from Illumina), two quantitative multiplex DNA reference standards (Tru-Q NGS DNA 3 and FFPE Quantitative Multiplex, Horizon Diagnostics, Cambridge, UK), and gDNA samples from Promega were included in each sequencing experiment for validation and contamination assessment.

### Validation of the NGS detected mutations

Conventional HRM analysis, Sanger sequencing and/or Taqman-MGB were used to validate new mutations detected by NGS, as previously described [38–42]. Some samples were also analyzed by NGS using the TruSight Tumor kit from Illumina according to the manufacturer's recommendations.

### Statistical analysis

Statistical analyses were carried out using Statgraphics Software. For correlation, a Pearson test was performed. P value less than 0.05 was considered to be statistically significant.

## SUPPLEMENTARY MATERIALS TABLES








