# Supplementary material for: High-throughput detection of clinically targetable alterations using next-generation sequencing
Source: Oncotarget. 2017 Mar 3;8(25):40345–58. doi: 10.18632/oncotarget.15875 (PMC5522202; doi:10.18632/oncotarget.15875)
Supplement: Supplementary file 1 [file oncotarget-08-40345-s001.pdf]

High-throughput detection of clinically targetable alterations using next-generation sequencing

SUPPLEMENTARY MATERIALS AND METHODS

Qualification of DNA extracted from FFPE samples

Amplifiability of the gDNA extracted from FFPE samples is determined by quantitative PCR (qPCR) using the KAPA SYBR FAST Master Mix Universal (Kapa Biosystems, Wilmington, USA). FFPE DNA and a quality control template (QCT), that is a non-FFPE reference gDNA, called, are analysed in triplicate. qPCR was performed on a LightCycler 480 (Roche, Meylan, France) using the PCR program detailed on the Illumina's procedure, with acquisition of signals after the elongation step. For each sample, the quantification cycle (Cq) is determined. The average Cq for the QCT was then subtracted from the average Cq for each sample to obtain the ΔQc for each sample. Samples were then diluted according to the ΔQc and the Illumina's recommandations as follow:

- Samples with a ΔQc from -2.5 to -1.5 were diluted 16-fold
- Samples with a ΔQc from -1.5 to -0.5 were diluted 8-fold
- Samples with a ΔQc from -0.5 to 0.5 were diluted 4-fold
- Samples with a ΔQc from 0.5 to 1.5 were diluted 2-fold
- Samples with a ΔQc from 1.5 to 4 were not diluted
- Samples with a ΔQc >4 that are normally not suitable for NGS analysis were not diluted

Filter criteria for variant calling

SNPs and short indels are identified using the somatic variant caller implemented in the SeqNext software (JSI Medical Systems, Ettenheim, Germany) according to the following criteria: (i) reads for which 60% of the bases have a quality score below Q20 were excluded from the analysis; (ii) only amplicons with a read depth of 100x or greater were used for variant calling; (iii) variants were called if they were present in both libraries with a mean VAF of 3% or greater.

SUPPLEMENTARY TABLES

Supplementary Table 1: Study of the inter-run reproducibility using the FFPE Quantitative Multiplex

Gene	Nucleotide change	Amino Acid change	VAF (Laboratory 2)			Expected VAF (%)[b]
			Mean[a]	SD	CV (%)	
EGFR	c.2369C>T	p.Thr790Met	0.53[c]	0.17	32.08	1.00
EGFR	c.2235_2249delGGA ATTAAGAGAAGC	p.Glu746_Ala750del	1.77	0.28	15.86	2.00
EGFR	c.2573T>G	p.Leu858Arg	3.14	0.50	15.90	3.00
KRAS	c.35G>A	p.Gly12Asp	6.08	0.05	0.82	6.00
MET	c.710delT	p.Leu238TyrfsTer25	7.30	0.33	4.56	6.50
PIK3CA	c.1633G>A	p.Glu545Lys	8.09	0.16	1.94	9.00
KIT	c.2447A>T	p.Asp816Val	8.81	0.48	5.44	10.00
BRAF	c.1799T>A	p.Val600Glu	12.01	0.32	2.66	10.50
NRAS	c.181C>A	p.Gln61Lys	13.94	1.06	7.60	12.50
KRAS	c.38G>A	p.Gly13Asp	14.65	1.19	8.14	15.00
PIK3CA	c.3140A>G	p.His1047Arg	16.97	0.29	1.70	17.50
EGFR	c.2155G>A	p.Gly719Ser	24.50	0.85	3.48	24.50

[a] Mean of three independent experiments
[b] Measured by ddPCR
[c] Mutations with a VAF below 3% were determined using IGV
SD, Standard deviation; CV, Coefficient of variation; VAF, Variance allele frequencies; ddPCR, droplet digital PCR

Supplementary Table 2: DSTP performance according to read depth

	Sample 1	Sample 2	Sample 3	Sample 4	Sample 5	Sample 6	Sample 7	Sample 8
Concentration (nM)	4	2	1	0.5	0.25	0.125	0.062	0.036
Number of true positive mutation detected	14	14	14	14	14	14	14	14
Number of false positive mutation detected	0	0	0	0	0	3	2	12
Number of amplicon with read depth <100	0	1	1	1	2	14	29	72
% of amplicons exluded	0	0,67	0,67	0,67	1,33	9,33	19,33	48,00
Precision (%)[a]	100	100	100	100	100	82,4	87,5	53,8
Median read depth	76424,5	6292,5	2732	1546,5	663,5	352	189	105
Minimal read depth	1553	67	26	14	18	11	9	5
Maximal read depth	183904	14787	6593	3253	1663	855	449	245

[a] Precision = TP/(TP+FP)*100

Supplementary Table 3: Description of the mutation detected using the DSTP and routine techniques in our tumor sample cohort.

See Supplementary File 1

Supplementary Table 4: Comparative results between the DSTP and the TruSight tumor panel for the Tru-Q NGS DNA3 control sample.

See Supplementary File 2

Supplementary Table 5: Comparative results between the DSTP and the TruSight tumor panel for FFPE clinical samples.

See Supplementary File 3
