# Supplementary material for: High-throughput detection of clinically targetable alterations using next-generation sequencing
Source: Oncotarget. 2017 Mar 3;8(25):40345–58. doi: 10.18632/oncotarget.15875 (PMC5522202; doi:10.18632/oncotarget.15875)
Supplement: Supplementary file 2 [file oncotarget-08-40345-s002.docx]

**Supplementary Table 3.** Description of the mutation detected using the DSTP and routine techniques in our tumor sample cohort

| **Sample ID** | **Tumor localisation** | **Tumor type** | **DCt** | **Gene** | **cDNA_change** | **Aa_change** | **Routine results** | **NGS results** | **% VAF** |
| --- | --- | --- | --- | --- | --- | --- | --- | --- | --- |
| C01 | Colorectal | Adenocarcinoma | -3.03 | *BRAF* | c.1799T>A | p.Val600Glu | Mutation detected | Mutation detected | 25.57 |
| C02 | Colorectal | Adenocarcinoma | 1.07 | *BRAF* | c.1799T>A | p.Val600Glu | Mutation detected | Mutation detected | 30.12 |
| C03 | Colorectal | Adenocarcinoma | 1.4 | *BRAF* | c.1799T>A | p.Val600Glu | Mutation detected | Mutation detected | 19.8 |
| C04 | Colorectal | Adenocarcinoma | 2.18 | *BRAF* | c.1799T>A | p.Val600Glu | Mutation detected | Mutation detected | 46.49 |
| C04 | Colorectal | Adenocarcinoma | 2.18 | *MET* | c.3718G>C | p.Asp1240His | Not explored | Mutation detected | 33.25 |
| C05 | Colorectal | Adenocarcinoma | 1.7 | *BRAF* | c.1742A>T | p.Asn581Ile | Not explored | Mutation detected | 24.83 |
| C05 | Colorectal | Adenocarcinoma | 1.7 | *ERBB4* | c.1181C>T | p.Thr394Ile | Not explored | Mutation detected | 34.46 |
| C05 | Colorectal | Adenocarcinoma | 1.7 | *FGFR3* | c.893T>C | p.Val298Ala | Not explored | Mutation detected | 35.8 |
| C05 | Colorectal | Adenocarcinoma | 1.7 | *KRAS* | c.57G>T | p.Leu19Phe | Not explored | Mutation detected | 33.71 |
| C06 | Colorectal | Adenocarcinoma | 1.08 | *KRAS* | c.34G>T | p.Gly12Cys | Mutation detected | Mutation detected | 57.27 |
| C07 | Colorectal | Adenocarcinoma | 5.39 | *KRAS* | c.35G>A | p.Gly12Asp | Mutation detected | Mutation detected | 26.53 |
| C08 | Colorectal | Adenocarcinoma | 5.08 | *KRAS* | c.38G>A | p.Gly13Asp | Mutation detected | Mutation detected | 40.74 |
| C08 | Colorectal | Adenocarcinoma | 5.08 | *MET* | c.1124A>G | p.Asn375Ser | Not explored | Mutation detected | 50.25 |
| C09 | Colorectal | Adenocarcinoma | 0.9 | *KRAS* | c.35G>A | p.Gly12Asp | Mutation detected | Mutation detected | 29.95 |
| C10 | Colorectal | Adenocarcinoma | 1.57 | *KRAS* | c.34G>A | p.Gly12Ser | Mutation detected | Mutation detected | 82.65 |
| C11 | Colorectal | Adenocarcinoma | 0.88 | *KRAS* | c.35G>C | p.Gly12Ala | Mutation detected | Mutation detected | 24.01 |
| C12 | Colorectal | Adenocarcinoma | 1.43 | *KRAS* | c.34G>A | p.Gly12Ser | Mutation detected | Mutation detected | 15.13 |
| C13 | Colorectal | Adenocarcinoma | 0.60 | *KRAS* | c.35G>T | p.Gly12Val | Mutation detected | Mutation detected | 37.91 |
| C14 | Colorectal | Adenocarcinoma | 2.16 | *KRAS* | c.38G>A | p.Gly13Asp | Mutation detected | Mutation detected | 42.8 |
| C15 | Colorectal | Adenocarcinoma | 6.56 | *KRAS* | c.35G>A | p.Gly12Asp | Mutation detected | Mutation detected | 44.48 |
| C16 | Colorectal | Adenocarcinoma | 0.65 | *KRAS* | c.35G>T | p.Gly12Val | Mutation detected | Mutation detected | 16.35 |
| C17 | Colorectal | Adenocarcinoma | 3.51 | *KRAS* | c.35G>T | p.Gly12Val | Mutation detected | Mutation detected | 25.15 |
| C17 | Colorectal | Adenocarcinoma | 3.51 | *MAP2K1* | c.199G>A | p.Asp67Asn | Not explored | Mutation detected | 31.52 |
| C18 | Colorectal | Adenocarcinoma | 1.7 | *KRAS* | c.35G>T | p.Gly12Val | Mutation detected | Mutation detected | 38.79 |
| C18 | Colorectal | Adenocarcinoma | 1.7 | *PIK3CA* | c.1633G>A | p.Glu545Lys | Not explored | Mutation detected | 24.71 |
| C19 | Colorectal | Adenocarcinoma | -0.38 | *KRAS* | c.38G>A | p.Gly13Asp | Mutation detected | Mutation detected | 18.31 |
| C19 | Colorectal | Adenocarcinoma | -0.38 | *PIK3CA* | c.3140A>G | p.His1047Arg | Not explored | Mutation detected | 18.14 |
| C20 | Colorectal | Adenocarcinoma | -3.67 | *KRAS* | c.35G>A | p.Gly12Asp | Mutation detected | Mutation detected | 30.28 |
| C20 | Colorectal | Adenocarcinoma | -3.67 | *PIK3CA* | c.1636C>A | p.Gln546Lys | Not explored | Mutation detected | 15.92 |
| C21 | Colorectal | Adenocarcinoma | 1.32 | *KRAS* | c.436G>A | p.Ala146Thr | Mutation detected | Mutation detected | 49.79 |
| C21 | Colorectal | Adenocarcinoma | 1.32 | *PIK3CA* | c.1633G>A | p.Glu545Lys | Not explored | Mutation detected | 26.54 |
| C22 | Colorectal | Adenocarcinoma | -2.19 | *EGFR* | c.2386G>A | p.Gly796Ser | Not explored | Mutation detected | 51.94 |
| C22 | Colorectal | Adenocarcinoma | -2.19 | *PIK3CA* | c.3119T>A | p.Met1040Lys | Not explored | Mutation detected | 40.04 |
| C23 | Colorectal | Adenocarcinoma | 3.65 | *FGFR3* | c.1150T>C | p.Phe384Leu | Not explored | Mutation detected | 56.15 |
| C24 | Colorectal | Adenocarcinoma | 0.9 | *MET* | c.1124A>G | p.Asn375Ser | Not explored | Mutation detected | 49.25 |
| C25 | Colorectal | Adenocarcinoma | 3.15 | *MET* | c.1124A>G | p.Asn375Ser | Not explored | Mutation detected | 59.16 |
| C26 | Colorectal | Adenocarcinoma | -0.45 | *MET* | c.1039G>A | p.Ala347Thr | Not explored | Mutation detected | 49.60 |
| C26 | Colorectal | Adenocarcinoma | -0.45 | *MET* | c.3029C>T | p.Thr1010Ile | Not explored | Mutation detected | 49.07 |
| C27 | Colorectal | Adenocarcinoma | -1.31 | WT |  |  | WT | WT |  |
| C28 | Colorectal | Adenocarcinoma | 1.3 | WT |  |  | WT | WT |  |
| C29 | Colorectal | Adenocarcinoma | 2.14 | WT |  |  | WT | WT |  |
| C30 | Colorectal | Adenocarcinoma | 5.44 | WT |  |  | WT | WT |  |
| C31 | Colorectal | Adenocarcinoma | 0.27 | WT |  |  | WT | WT |  |
| C32 | Colorectal | Adenocarcinoma | -1.03 | WT |  |  | WT | WT |  |
| C33 | Colorectal | Adenocarcinoma | -0.63 | WT |  |  | WT | WT |  |
| C34 | Colorectal | Adenocarcinoma | 0.74 | WT |  |  | WT | WT |  |
| C35 | Colorectal | Adenocarcinoma | 1.72 | WT |  |  | WT | WT |  |
| C36 | Colorectal | Adenocarcinoma | 0.61 | WT |  |  | WT | WT |  |
| C37 | Colorectal | Adenocarcinoma | 0.4 | WT |  |  | WT | WT |  |
| C38 | Colorectal | Adenocarcinoma | 3.48 | WT |  |  | WT | WT |  |
| C39 | Colorectal | Adenocarcinoma | -1.5 | WT |  |  | WT | WT |  |
| C40 | Colorectal | Adenocarcinoma | 0.25 | WT |  |  | WT | WT |  |
| C41 | Colorectal | GIST | 1.99 | *EGFR* | c.2230A>G | p.Ile744Val | Not explored | Mutation detected | 48.79 |
| C41 | Colorectal | GIST | 1.99 | *KIT* | c.1669T>C | p.Trp557Arg | Mutation detected | Mutation detected | 16.61 |
| C42 | Colorectal | GIST | 0.16 | *KIT* | c.1671_1685delGAAGGTTGTTGAGGA | p.Lys558_Glu562del | Mutation detected | Mutation detected | 67.42 |
| C43 | Colorectal | GIST | -1.15 | *KIT* | c.1743-1744 ins 27 | p.K581_W582ins | Mutation detected | Mutation detected | 20 |
| C44 | Colorectal | GIST | -2.48 | *KIT* | c.1656_1667delGTATGAAGTACA | p.Tyr553_Gln556del | Mutation detected | Mutation detected | 38.39 |
| C45 | Colorectal | GIST | 3.72 | *KIT* | c.1669T>A | p.Trp557Arg | Mutation detected | Mutation detected | 25.28 |
| C46 | Colorectal | GIST | -0.57 | *KIT* | c.1676T>G | p.Val559Gly | Mutation detected | Mutation detected | 77.12 |
| L01 | Lung | Adenocarcinoma | 5.35 | *EGFR* | c.2235_2249delGGAATTAAGAGAAGC | p.Glu746_Ala750del | Mutation detected | Mutation detected | 51.61 |
| L02 | Lung | Adenocarcinoma | 5.84 | *EGFR* | c.2573T>G | p.Leu858Arg | Mutation detected | Mutation detected | 77.44 |
| L03 | Lung | Adenocarcinoma | 5.87 | *EGFR* | c.2369C>T | p.Thr790Met | Mutation detected | Mutation detected | 32.1 |
| L03 | Lung | Adenocarcinoma | 5.87 | *EGFR* | c.2573T>G | p.Leu858Arg | Mutation detected | Mutation detected | 91.75 |
| L04 | Lung | Adenocarcinoma | 4.47 | *EGFR* | c.2236_2250delGAATTAAGAGAAGCA | p.Glu746_Ala750delinsdel | Mutation detected | Mutation detected | 47.2 |
| L05 | Lung | Adenocarcinoma | 4.49 | *EGFR* | c.2573T>G | p.Leu858Arg | Mutation detected | Mutation detected | 47.66 |
| L06 | Lung | Adenocarcinoma | -3.61 | *AKT1* | c.143G>A | p.Arg48His | Not explored | Mutation detected | 22.66 |
| L06 | Lung | Adenocarcinoma | -3.61 | *KRAS* | c.35G>T | p.Gly12Val | Mutation detected | Mutation detected | 31.52 |
| L07 | Lung | Adenocarcinoma | -0.11 | *BRAF* | c.1397G>T | p.Gly466Val | Not explored | Mutation detected | 6.47 |
| L07 | Lung | Adenocarcinoma | -0.11 | *KRAS* | c.37G>T | p.Gly13Cys | Mutation detected | Mutation detected | 7.56 |
| L08 | Lung | Adenocarcinoma | 7.48 | *FGFR3* | c.1150T>C | p.Phe384Leu | Not explored | Mutation detected | 23.83 |
| L08 | Lung | Adenocarcinoma | 7.48 | *KRAS* | c.182A>T | p.Gln61Leu | Mutation detected | Mutation detected | 23.52 |
| L09 | Lung | Adenocarcinoma | -0.91 | *KRAS* | c.34G>T | p.Gly12Cys | Mutation detected | Mutation detected | 35.76 |
| L10 | Lung | Adenocarcinoma | 0.49 | *KRAS* | c.183A>T | p.Gln61His | Mutation detected | Mutation detected | 71.1 |
| L11 | Lung | Adenocarcinoma | -0.88 | *KRAS* | c.34G>T | p.Gly12Cys | Mutation detected | Mutation detected | 5.13 |
| L12 | Lung | Adenocarcinoma | 1.89 | *KRAS* | c.35G>A | p.Gly12Asp | Mutation detected | Mutation detected | 19.4 |
| L13 | Lung | Adenocarcinoma | 3.57 | *KRAS* | c.34G>T | p.Gly12Cys | Mutation detected | Mutation detected | 19.20 |
| L14 | Lung | Adenocarcinoma | 0.59 | *KRAS* | c.37G>T | p.Gly13Cys | Mutation detected | Mutation detected | 24.03 |
| L15 | Lung | Adenocarcinoma | -1.76 | *KRAS* | c.34G>T | p.Gly12Cys | Mutation detected | Mutation detected | 35.46 |
| L16 | Lung | Adenocarcinoma | 0.12 | *KRAS* | c.35G>T | p.Gly12Val | Mutation detected | Mutation detected | 67.50 |
| L17 | Lung | Adenocarcinoma | 8.15 | *KRAS* | c.35G>C | p.Gly12Ala | Mutation detected | Mutation detected | 12.28 |
| L18 | Lung | Adenocarcinoma | 0.1 | *KRAS* | c.34G>T | p.Gly12Cys | Mutation detected | Mutation detected | 26.07 |
| L19 | Lung | Adenocarcinoma | -0.49 | *KRAS* | c.35G>T | p.Gly12Val | Mutation detected | Mutation detected | 32.07 |
| L19 | Lung | Adenocarcinoma | -0.49 | *MAP2K1* | c.169A>G | p.Lys57Glu | Not explored | Mutation detected | 32.63 |
| L20 | Lung | Adenocarcinoma | 0.51 | *FGFR2* | c.1639A>G | p.Ile547Val | Not explored | Mutation detected | 47.64 |
| L21 | Lung | Adenocarcinoma | 6.38 | *FGFR3* | c.772G>A | p.Gly258Arg | Not explored | Mutation detected | 9.20 |
| L22 | Lung | Adenocarcinoma | 0.8 | *FGFR3* | c.820C>T | p.His274Tyr | Not explored | Mutation detected | 6.00 |
| L23 | Lung | Adenocarcinoma | 0.29 | *FGFR3* | c.1150T>C | p.Phe384Leu | Not explored | Mutation detected | 45.22 |
| L24 | Lung | Adenocarcinoma | -5.51 | *FGFR3* | c.797T>G | p.Val266Gly | Not explored | Mutation detected | 5.22 |
| L25 | Lung | Adenocarcinoma | -3.61 | *HRAS* | c.269T>C | p.Phe90Ser | Not explored | Mutation detected | 48.01 |
| L26 | Lung | Adenocarcinoma | 1.05 | *KIT* | c.1339G>C | p.Glu447Gln | Not explored | Mutation detected | 28.74 |
| L27 | Lung | Adenocarcinoma | -0.98 | *MAP2K1* | c.171G>T | p.Lys57Asn | Not explored | Mutation detected | 40.71 |
| L28 | Lung | Adenocarcinoma | 3.11 | *MET* | c.1124A>G | p.Asn375Ser | Not explored | Mutation detected | 66.07 |
| L29 | Lung | Adenocarcinoma | 0.28 | *MET* | c.1124A>G | p.Asn375Ser | Not explored | Mutation detected | 75.79 |
| L30 | Lung | Adenocarcinoma | 1.55 | *MET* | c.3337G>T | p.Gly1113Trp | Not explored | Mutation detected | 25.12 |
| L31 | Lung | Adenocarcinoma | 0.51 | *MET* | c.2987C>T | p.Pro996Leu | Not explored | Mutation detected | 14.6 |
| L32 | Lung | Adenocarcinoma | 5.51 | WT |  |  | WT | WT |  |
| L33 | Lung | Adenocarcinoma | 2.81 | WT |  |  | WT | WT |  |
| L34 | Lung | Adenocarcinoma | 0.36 | WT |  |  | WT | WT |  |
| L35 | Lung | Adenocarcinoma | 0.63 | WT |  |  | WT | WT |  |
| L36 | Lung | Adenocarcinoma | 5.41 | WT |  |  | WT | WT |  |
| L37 | Lung | Adenocarcinoma | -0.06 | WT |  |  | WT | WT |  |
| L38 | Lung | Adenocarcinoma | -1.65 | WT |  |  | WT | WT |  |
| L39 | Lung | Adenocarcinoma | -4.57 | WT |  |  | WT | WT |  |
| L40 | Lung | Adenocarcinoma | 1.32 | WT |  |  | WT | WT |  |
| L41 | Lung | Adenocarcinoma | 1.53 | WT |  |  | WT | WT |  |
| L42 | Lung | Adenocarcinoma | 0.81 | WT |  |  | WT | WT |  |
| L43 | Lung | Adenocarcinoma | -0.35 | WT |  |  | WT | WT |  |
| L44 | Lung | Adenocarcinoma | 2.84 | WT |  |  | WT | WT |  |
| L45 | Lung | Adenocarcinoma | 3.44 | WT |  |  | WT | WT |  |
| L46 | Lung | Adenocarcinoma | 2.48 | WT |  |  | WT | WT |  |
| L47 | Lung | Adenocarcinoma | 2.03 | WT |  |  | WT | WT |  |
| L48 | Lung | Adenocarcinoma | 4.25 | WT |  |  | WT | WT |  |
| L49 | Lung | Adenocarcinoma | 3.17 | WT |  |  | WT | WT |  |
| L50 | Lung | Adenocarcinoma | 1.23 | WT |  |  | WT | WT |  |
| L51 | Lung | Adenocarcinoma | 3.31 | WT |  |  | WT | WT |  |
| L52 | Lung | Adenocarcinoma | 4.74 | WT |  |  | WT | WT |  |
| L53 | Lung | Adenocarcinoma | 2.88 | WT |  |  | WT | WT |  |
| L54 | Lung | Adenocarcinoma | 5.63 | WT |  |  | WT | WT |  |
| L55 | Lung | Adenocarcinoma | 3.84 | WT |  |  | WT | WT |  |
| L56 | Lung | Adenocarcinoma | 4.55 | WT |  |  | WT | WT |  |
| L57 | Lung | Adenocarcinoma | 5.88 | WT |  |  | WT | WT |  |
| L58 | Lung | Adenocarcinoma | 2.32 | WT |  |  | WT | WT |  |
| L59 | Lung | Adenocarcinoma | 1.38 | WT |  |  | WT | WT |  |
| L60 | Lung | Adenocarcinoma | 10.05 | WT |  |  | WT | WT |  |
| L61 | Lung | Adenocarcinoma | 0.86 | WT |  |  | WT | WT |  |
| L62 | Lung | Adenocarcinoma | 2.4 | WT |  |  | WT | WT |  |
| L63 | Lung | Adenocarcinoma | 0.4 | WT |  |  | WT | WT |  |
| L64 | Lung | Adenocarcinoma | 0.7 | WT |  |  | WT | WT |  |
| L65 | Lung | Adenocarcinoma | 6.5 | WT |  |  | WT | WT |  |
| L66 | Lung | Adenocarcinoma | 0.23 | WT |  |  | WT | WT |  |
| L67 | Lung | Adenocarcinoma | 1.04 | WT |  |  | WT | WT |  |
| L68 | Lung | Adenocarcinoma | 5.485 | WT |  |  | WT | WT |  |
| S01 | Skin | Melanoma | 5.87 | *BRAF* | c.1799T>A | p.Val600Glu | Mutation detected | Mutation detected | 52.78 |
| S02 | Skin | Melanoma | 5.19 | *BRAF* | c.1799T>A | p.Val600Glu | Mutation detected | Mutation detected | 56.86 |
| S03 | Skin | Melanoma | 0.75 | *BRAF* | c.1798_1799delGTinsAA | p.Val600Lys | Mutation detected | Mutation detected | 30.67 |
| S04 | Skin | Melanoma | 3.98 | *BRAF* | c.1798_1799delGTinsAG | p.Val600Arg | Mutation detected | Mutation detected | 20.19 |
| S05 | Skin | Melanoma | 1.86 | *BRAF* | c.1799_1800delTGinsAA | p.Val600Glu | Mutation detected | Mutation detected | 37.87 |
| S06 | Skin | Melanoma | 5.04 | *BRAF* | c.1400C>T | p.Ser467Leu | Not explored | Mutation detected | 19.76 |
| S06 | Skin | Melanoma | 5.04 | *ERBB2* | c.2456C>T | p.Ser819Phe | Not explored | Mutation detected | 18.83 |
| S06 | Skin | Melanoma | 5.04 | *KRAS* | c.35G>A | p.Gly12Asp | Not explored | Mutation detected | 18.2 |
| S07 | Skin | Melanoma | 3.99 | *EGFR* | c.2569_2570delGGinsAA | p.Gly857Lys | Not explored | Mutation detected | 37.06 |
| S07 | Skin | Melanoma | 3.99 | *NRAS* | c.182A>T | p.Gln61Leu | Mutation detected | Mutation detected | 55.69 |
| S08 | Skin | Melanoma | 3.8 | *NRAS* | c.182A>G | p.Gln61Arg | Mutation detected | Mutation detected | 15.00 |
| S09 | Skin | Melanoma | 1.72 | *NRAS* | c.182A>G | p.Gln61Arg | Mutation detected | Mutation detected | 38.89 |
| S10 | Skin | Melanoma | 0.86 | *NRAS* | c.385C>T | p.Gln129* | Not explored | Mutation detected | 22.37 |
| S11 | Skin | Melanoma | 1.4 | *MET* | c.504G>T | p.Glu168Asp | Not explored | Mutation detected | 48.07 |
| S12 | Skin | Melanoma | 6.03 | WT |  |  | WT | WT |  |
| S13 | Skin | Melanoma | 1.68 | WT |  |  | WT | WT |  |
| S14 | Skin | Melanoma | 0.3 | WT |  |  | WT | WT |  |
| S15 | Skin | Melanoma | 7.59 | WT |  |  | WT | WT |  |
| S16 | Skin | Melanoma | 0.77 | WT |  |  | WT | WT |  |

WT, Wild-type
