# Supplementary material for: High-throughput detection of clinically targetable alterations using next-generation sequencing
Source: Oncotarget. 2017 Mar 3;8(25):40345–58. doi: 10.18632/oncotarget.15875 (PMC5522202; doi:10.18632/oncotarget.15875)
Supplement: Supplementary file 3 [file oncotarget-08-40345-s003.docx]

**Supplementary Table 4.** Comparative results between the DSTP and the TruSight tumor panel for the Tru-Q NGS DNA3 control sample

| DSTP | | | | TruSight Tumor | | | |
| --- | --- | --- | --- | --- | --- | --- | --- |
| Gene | Nucleotide change | Aa change | % VAF | Gene | Nucleotide change | Aa change | % VAF |
| *BRAF* | c.1798G>A | p.Val600Met | 3.88 | *BRAF* | c.1798G>A | p.Val600Met | 3.92 |
| *BRAF* | c.1799T>A | p.Val600Glu | 7.57 | *BRAF* | c.1799T>A | p.Val600Glu | 8.28 |
| *EGFR* | c.2235_2249delGGAATTAAGAGAAGC | p.Glu746_Ala750del | 4.13 | *EGFR* | c.2235_2249delGGAATTAAGAGAAGC | p.Glu746_Ala750del | 3.81 |
| *EGFR* | c.2155G>A | p.Gly719Ser | 16.40 | *EGFR* | c.2155G>A | p.Gly719Ser | 14.88 |
| *KRAS* | c.436G>A | p.Ala146Thr | 4.43 | *KRAS* | c.436G>A | p.Ala146Thr | 6.04 |
| *KRAS* | c.34G>A | p.Gly12Ser | 4.78 | *KRAS* | c.34G>A | p.Gly12Ser | 4.84 |
| *KRAS* | c.38G>A | p.Gly13Asp | 25.13 | *KRAS* | c.38G>A | p.Gly13Asp | 25.26 |
| *MET* | c.3757T>G | p.Tyr1253Asp | 4.25 | *MET* | c.3757T>G | p.Tyr1253Asp | 3.82 |
| *MET* | c.710delT | p.Leu238TyrfsTer25 | 20.33 | *MET* | c.710delT | p.Leu238TyrfsTer25 | 18.17 |
| *NRAS* | c.183A>T | p.Gln61His | 4.42 | *NRAS* | c.183A>T | p.Gln61His | 5.89 |
| *PIK3CA* | c.3140A>G | p.His1047Arg | 29.09 | *PIK3CA* | c.3140A>G | p.His1047Arg | 31.95 |
| *PIK3CA* | c.1624G>A | p.Glu542Lys | 5.07 | Not targeted by the panel | | | |
