# Supplementary material for: High-throughput detection of clinically targetable alterations using next-generation sequencing
Source: Oncotarget. 2017 Mar 3;8(25):40345–58. doi: 10.18632/oncotarget.15875 (PMC5522202; doi:10.18632/oncotarget.15875)
Supplement: Supplementary file 4 [file oncotarget-08-40345-s004.docx]

**Supplementary Table 5.** Comparative results between the DSTP and the TruSight tumor panel for FFPE clinical samples

|  | DSTP | | | | TruSight Tumor | | | |
| --- | --- | --- | --- | --- | --- | --- | --- | --- |
| Sample ID | Gene | Nucleotide change | Aa change | % VAF | Gene | Nucleotide change | Aa change | % VAF |
| L34 | WT | | | | WT | | | |
| L23 | *MAP2K1* | c.171G>T | p.Lys57Asn | 40.71 | *MAP2K1* | c.171G>T | p.Lys57Asn | 40.43 |
| L15 | *KRAS* | c.35G>T | p.Gly12Val | 67.50 | *KRAS* | c.35G>T | p.Gly12Val | 70.20 |
| L21 | *HRAS* | c.269T>C | p.Phe90Ser | 48.01 | Not targeted by the panel | | | |
| L35 | Not targeted by the panel | | | | *TP53* | c.743G>A | p.Arg248Gln | 47.40 |

WT. Wild-type
